# Secondary Post-Geniculate Involvement in Leber’s Hereditary Optic Neuropathy

**DOI:** 10.1371/journal.pone.0050230

**Published:** 2012-11-27

**Authors:** Giovanni Rizzo, Kevin R. Tozer, Caterina Tonon, David Manners, Claudia Testa, Emil Malucelli, Maria Lucia Valentino, Chiara La Morgia, Piero Barboni, Ruvdeep S. Randhawa, Fred N. Ross-Cisneros, Alfredo A. Sadun, Valerio Carelli, Raffaele Lodi

**Affiliations:** 1 Department of Biomedical and NeuroMotor Sciences (DiBiNeM), University of Bologna, Bologna, Italy; 2 “IRCCS Istituto delle Scienze Neurologiche”, Bologna, Italy; 3 Doheny Eye Institute and Department of Ophthalmology, Keck School of Medicine, University of Southern California, Los Angeles, California, United States of America; 4 Studio Oculistico d’Azeglio, Bologna, Italy; Charité University Medicine Berlin, Germany

## Abstract

Leber’s hereditary optic neuropathy (LHON) is characterized by retinal ganglion cell (RGC) degeneration with the preferential involvement of those forming the papillomacular bundle. The optic nerve is considered the main pathological target for LHON. Our aim was to investigate the possible involvement of the post-geniculate visual pathway in LHON patients. We used diffusion-weighted imaging for in vivo evaluation. Mean diffusivity maps from 22 LHON visually impaired, 11 unaffected LHON mutation carriers and 22 healthy subjects were generated and compared at level of optic radiation (OR). Prefrontal and cerebellar white matter were also analyzed as internal controls. Furthermore, we studied the optic nerve and the lateral geniculate nucleus (LGN) in post-mortem specimens obtained from a severe case of LHON compared to an age-matched control. Mean diffusivity values of affected patients were higher than unaffected mutation carriers (P<0.05) and healthy subjects (P<0.01) in OR and not in the other brain regions. Increased OR diffusivity was associated with both disease duration (B = 0.002; P<0.05) and lack of recovery of visual acuity (B = 0.060; P<0.01). Post-mortem investigation detected atrophy (41.9% decrease of neuron soma size in the magnocellular layers and 44.7% decrease in the parvocellular layers) and, to a lesser extent, degeneration (28.5% decrease of neuron density in the magnocellular layers and 28.7% decrease in the parvocellular layers) in the LHON LGN associated with extremely severe axonal loss (99%) in the optic nerve. The post-geniculate involvement in LHON patients is a downstream post-synaptic secondary phenomenon, reflecting de-afferentation rather than a primary neurodegeneration due to mitochondrial dysfunction of LGN neurons.

## Introduction

Leber’s hereditary optic neuropathy (LHON) is a mitochondrial disease characterized by retinal ganglion cells (RGCs) degeneration due to maternally inherited point mutations in mitochondrial DNA (mtDNA) that affect the respiratory complex I [1], [2]. Characteristically, the degenerative process preferentially involves the RGCs forming the papillomacular bundle serving central vision, colour vision and high spatial frequency contrast sensitivity [3]. LHON affects prevalently young males, who suffer an acute/subacute loss of central vision that leads to rapid decrease of visual acuity due to central scotoma. This acute phase consolidates in a chronic state in about one year after the onset of visual loss, leaving the patients with optic atrophy and usually permanent blindness [1], [2].

However, some of the patients may experience various degrees of visual function recovery, with gain of visual acuity and shrinkage or fenestration of the central scotoma at visual field [1], [2]. This recovery may occur spontaneously, most frequently with one of the common mutations at position 14484/ND6 and if the age at disease onset is precocious, irrespectively the mutation type. Recently, it has been demonstrated that administration of idebenone may also increase the rate of visual function recovery [4], [5]. Long-lasting chronic cases may suffer further slow rate loss of RGCs, as documented by a few cases studied post-mortem, supporting a long-range neurodegenerative activity [1], [3].

Most of the mutation carriers along the maternal line remain, however, unaffected indicating that the mtDNA pathogenic mutation is a necessary but not sufficient condition to develop the optic neuropathy. Even in the absence of visual loss, the unaffected mutation carriers may display subclinical changes at fundus exam [6], neurophysiological and optical coherence tomography investigations [7], [8], as well as bioenergetic impairment measured by biochemical testing and by *in-vivo* magnetic resonance spectroscopy [9].

LHON is, along with dominant optic atrophy (DOA), a non-syndromic mitochondrial optic neuropathy, both being characterized by visual loss and optic nerve atrophy as the only or at least prevalent pathological feature [1], [2]. Optic nerve involvement may also occur in more complex syndromes due to mitochondrial dysfunction such as Friedreich’s ataxia (FRDA), in which diffusion-weighted imaging (DWI) studies have demonstrated an involvement not only of the optic nerve but also of the retro-geniculate optic radiation (OR) [10], [11]. Recently, two studies applying voxel-based morphometry (VBM) and DTI approaches, pointed towards OR abnormalities in LHON patients, suggesting trans-synaptic degeneration without definitely excluding a primary dysfunction [12], [13]. This was suggested by previous phosphorus MR spectroscopy (^31^P-MRS) investigations showing occipital lobes bioenergetics dysfunction in LHON patients and unaffected mutation carriers [9], [14]–[16].

The aim of the present study was to better characterize the post-geniculate involvement in LHON. We used DWI, a technique demonstrated to disclose increased water diffusivity in the brain areas where atrophy and/or gliosis occur [17], to investigate the optic radiations in LHON patients and unaffected mutation carriers. Furthermore, we studied the lateral geniculate nucleus (LGN) in post-mortem specimens, comparing average neuron cell body size and cell density between a LHON patient with severe axonal loss in the optic nerve and an age-matched control.

## Methods

### Subjects

Twenty-two molecularly-certified LHON affected patients (17 males; age 33±11, mean ± SD), 11 unaffected mutation carriers (5 males; age 45±15) and 22 healthy controls (16 males; age 37±17) were studied between February 2006 and December 2009 at the Functional MR Unit, University of Bologna, Italy. Exclusion criteria were presence of neurological symptoms or signs not due to the optic atrophy and evidence of grey/white matter alterations on conventional MRI scanning. None of unaffected mutation carriers and controls had a history of neurologic or psychiatric diseases and conventional MRI scans appeared normal in all cases. All participants gave their written informed consent and the study was approved by the institutional review board of the Bologna Hospital.

### MR Examination

Subjects were studied in a 1.5 T General Electrics Medical Systems (Milwaukee, Wisconsin) Signa Horizon LX whole-body scanner. Structural imaging included T1- and T2-weighted fast spin-echo scans. Axial DW images were obtained (slice thickness = 5 mm, inter-slice gap = 1 mm) using a single-shot EPI sequence (matrix size = 192×192). Orthogonal x, y and z diffusion-encoding gradients were applied with gradient strengths corresponding to b-values of 300, 600 and 900 s/mm^2^. In addition, images without diffusion weighting were acquired, corresponding to b = 0 s/mm^2^ and exhibiting T2-contrast. The total DWI scan time was 2 min.

**Figure 1:**
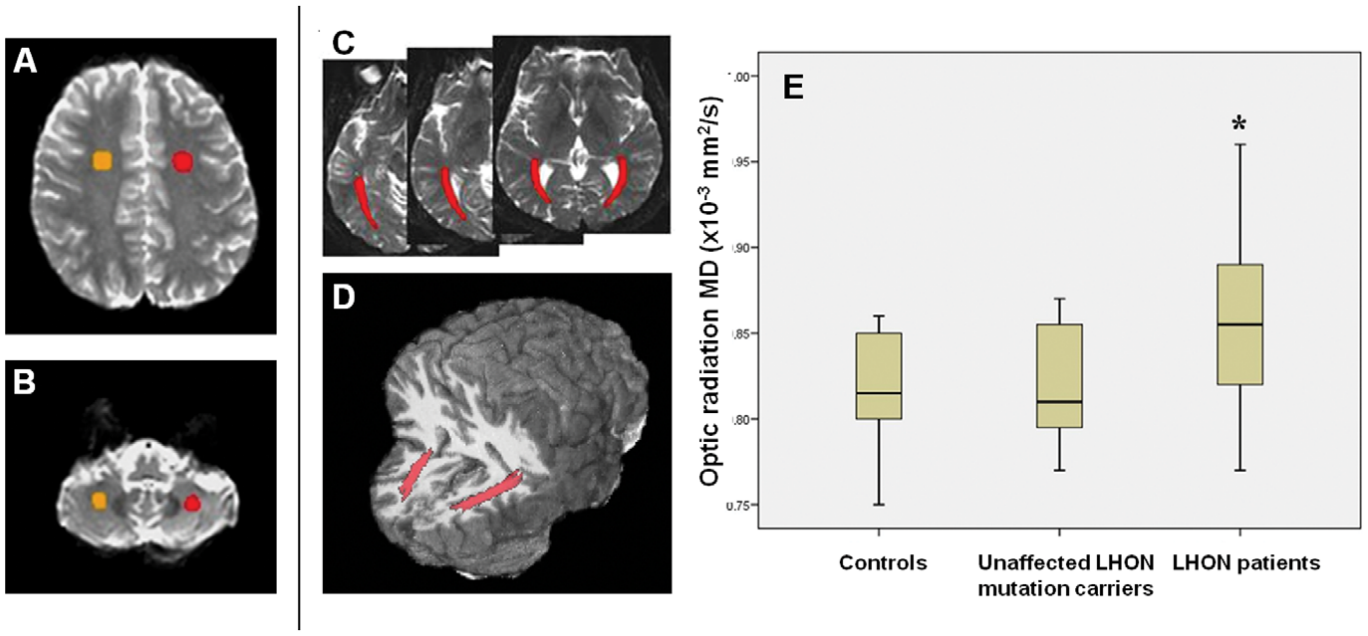
Segmentation of ROIs including prefrontal white matter (A), cerebellar white matter (B) and optic radiation (C) on axial T2 images. D: 3D reconstruction of optic radiation ROIs on a registered T1 volumetric image. E: Box-plot of MD values of optic radiations in controls, LHON healthy carriers and LHON patients. (Each box shows the median, quartiles, extreme values; * = P<0.01).

Distortions in the DW-EPI images due to gradient-induced eddy currents were corrected using the image registration software FLIRT (www.fmrib.ox.ac.uk/fsl). Due to the nature of the distortions, the degrees of freedom were restricted to translation, scaling, and shearing along the phase encoding direction [18]. Possible head movements were corrected using image registration of each volume to the first restricting degrees of freedom to translation and rotation. Mean diffusivity (MD) was determined pixel-wise using a least-squares fit using the program DTIFIT. In order to avoid contamination of the MD values for grey and white matter by the much higher values of cerebral spinal fluid (CSF) during further evaluation, pixels containing CSF were masked from the MD map. This was accomplished using the FAST algorithm for a two-class segmentation based on the corresponding T2-weighted EPI images. Regions of interest (ROIs) **(** **Fig. 1A–B** **)** were determined by segmentation of the left and right ORs on three slices using the T2-weighted EPI images and were superimposed on the MD maps to obtain mean MD values. We also delineated ROIs at the level of the prefrontal and cerebellar white matter, as internal controls.

### Pathological Examination

The postmortem lateral geniculate nuclei (LGN) from a previously studied 75 year-old female LHON patient [3] and a 75 year old female control individual were analysed at the Doheny Eye Institute, Los Angeles, California. The control brain was acquired from the Alzheimer’s Disease Research Center at the University of Southern California, having no evidence of either Alzheimer’s Disease or other neurodegenerative diseases at the pathological report. The LHON patient had an extremely severe chronic optic neuropathy and carried the homoplasmic 3460G>A mutation. The LGNs for both cases were taken from fixed brains in neutral buffered formalin, dissected in a coronal plane, processed for and embedded into paraffin blocks, sectioned, and placed on glass microscope slides. The tissue sections were stained with hematoxylin for Nissl substance to allow for identification of the cell bodies and coverslipped using permanent mounting media.

**Figure 2:**
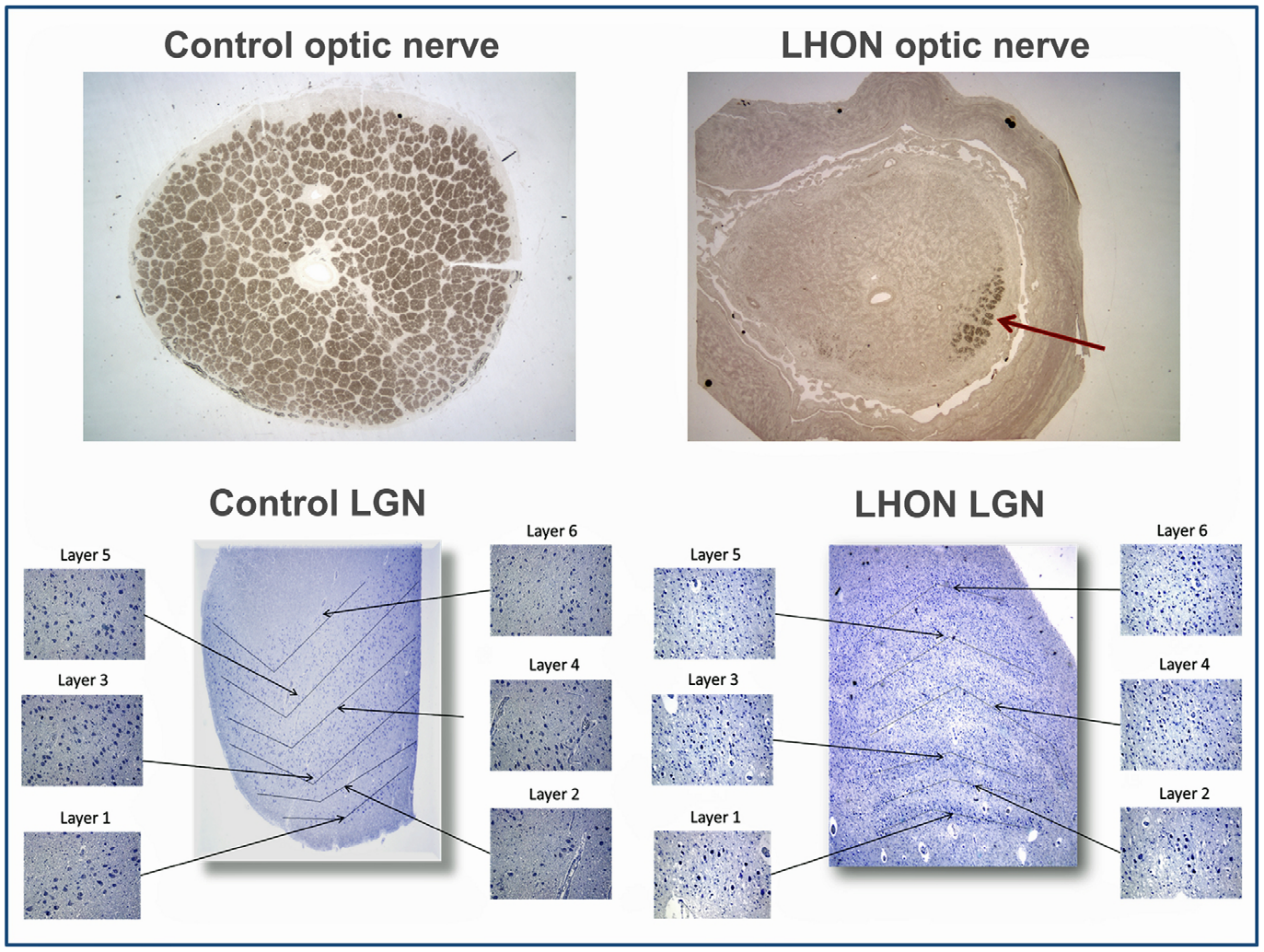
Top panel: optic nerves in cross-section and stained by p-Phenylenediamine for control and LHON patient (25x magnification). In the LHON patient only a small patch of fibers remains (arrow) in the super-nasal quadrant. Bottom panel: lateral geniculate nuclei (LGN) of control and LHON patients with all magnocellular (1 and 2) and parvocellular (3–6) layers identified (25x magnification). Insets represent samples of each zone at 200x magnification.

Image acquisition was performed using the T3 Aperio C3 digital scanning microscope (Aperio Corporation, Vista, CA) with the ImageScope analysis software package. The two LGN slides were digitized by the microscope using a 40x objective, which provides a 0.25 µm per pixel scanning resolution [19]. In each digitized LGN the two magnocellular and four parvocellular layers were identified **(** **Figure 2** **bottom panel)**. The six layers were each further subdivided into nasal, middle, and temporal zones for analysis purposes providing a total of 18 separate areas. The average cell size was then calculated using a custom algorithm built in the ImageScope software package designed to detect all areas that stained blue with hematoxylin. Only areas of positive staining that were greater than 100 µm^2^ were counted, as smaller areas likely represented intergeniculate or glial cell bodies and not neuronal cell bodies^­^. Following the software analysis, each section was further examined by the same observer for confirmation. Cell bodies that were not counted or counted in error by the software were manually corrected.

Postmortem optic nerves were fixed in a mixture of buffered glutaraldehyde/paraformaldehyde, dissected into cross-sections from both the LHON patient and the control subject. The tissues were then processed for and embedded into plastic, sectioned, and stained with p-Phenylenediamine (PPD) **(** **Figure 2** **, top panel)**. Myelinated axonal profiles were counted as previously detailed [3], [20].

### Statistical Analysis

Statistical analyses were performed using SPSS 14.0 for Windows. Parametric tests were used as Kolmogorov–Smirnov testing showed that the variables were normally distributed. One-way analysis of variance (ANOVA) followed by a post-hoc LSD test was used for comparison between groups. P values less than 0.05 after Bonferroni correction for multiple comparisons were accepted as statistically significant. To investigate the effect of genetic, demographic and clinical parameters (gender, type of mutation, age at onset, disease duration, and history of recovery of visual acuity) on DWI data, we used a general linear model (GLM) in which mean optic radiation diffusivity was the dependent variable, the categorical variables were defined as random factors and continuous variables as covariates, without interactions. For pathological examination we performed a descriptive analysis.

## Results

### Clinical and Genetic Features

All patients had the typical clinical and ophthalmological features of LHON. Fourteen had the G11778A/ND4, seven the G3460A/ND1, and one the T14484C/ND6 homoplasmic mutations. Six patients (two with 11778/ND4, three with 3460/ND1 and one with 14484/ND6 mutation) had a history of bilateral recovery of visual acuity (current visual acuity ranged from 0.125 to 1) whereas the remaining cases did not (current visual acuity ranged from 0.0025 to 0.075). Patients were considered to have recovered visual acuity after a gain of at least two lines on Snellen acuity or a change from “off chart” to “on chart”, as previously established [21], [22]. All patients were administered with idebenone as previously reported [5]. Age at onset ranged from 2 to 44 years (mean = 20 years), and disease duration from 1 to 35 years (mean = 14 years). Unaffected LHON mutation carriers (eight with the 11778/ND4 and three with the 3460/ND1 mutation) did not present any visual or neurological symptoms and had normal visual acuity.

### MR Findings

Conventional MRI did not demonstrate abnormalities in both the LHON patients and carriers.

In DWI analysis right- and left-side MD values were not statistically different for all ROIs and are reported as mean. One-way ANOVA detected a group difference (F = 6.8; P<0.01) only in ORs and post hoc testing revealed an increase in OR MD of LHON patients compared with both unaffected LHON mutation carriers (P<0.05) and healthy subjects (P<0.01) **(** **Fig. 1C** **)**. Optic radiation MD values were similar in unaffected mutation carriers and healthy subjects. In LHON patients GLM analysis disclosed that lack of visual acuity recovery (B = 0.060; P<0.01) and disease duration (B = 0.002; P<0.05) were significantly associated with increased OR MD values **(** **Table 1** **)**.

**Table 1:** MD values of optic radiation, prefrontal white matter and cerebellar white matter in LHON patients, LHON healthy carriers and controls with group comparison results (first two sections).

One-way ANOVA					
ROI*	LHON patients MD(×10^−3^ mm^2^/s)	LHON carriers MD(×10^−3^ mm^2^/s)		Controls MD(×10^−3^ mm^2^/s)	*^P#^*
Optic radiation	0.86±0.04	0.82±0.04		0.82±0.03	*<0.01*
Prefrontal WM	0.75±0.04	0.75±0.02		0.77±0.04	n.s.
Cerebellar WM	0.73±0.04	0.73±0.05		0.74±0.05	n.s.
**Post-hoc LSD test for optic radiation MD values**					
LHON patients *vs* healthy controls					*<0.01*
LHON patients *vs* unaffected LHON carriers					*<0.05*
unaffected LHON carriers *vs* healthy controls					n.s.
**GLM** (dependent variable: optic radiation MD values in LHON patients)					
Disease duration			*B = 0.002; P = <0.05*		
Lack of recovery of visual acuity			*B = 0.06; P = <0.01*		

The bottom of the table shows the results of GLM analysis used to evaluate the effect of genetic, clinical and demographic data on optic radiation MD values in LHON patients.

* = mean of left and right MD values.

# = corrected for multiple comparisons.

Values are reported as mean and standard deviation.

MD: mean diffusivity; WM: white matter; n.s.: not significant; GLM: general linear model.

### Pathological Findings

**Figure 3:**
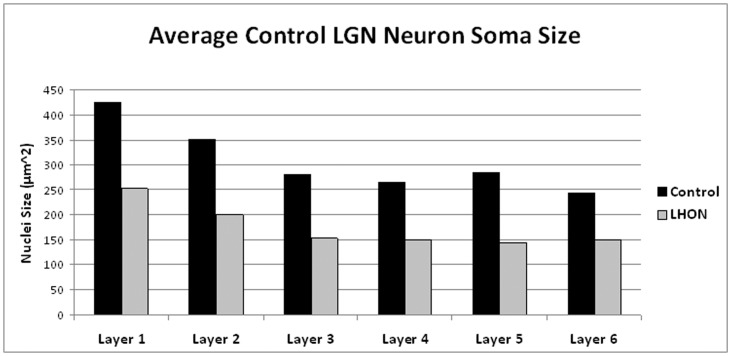
Average neuron soma size of all layers in the LGNs from the LHON patient and the age-matched control.

Pathologic examination of the LGNs from the LHON patient showed a marked decrease in the average neuron soma across all six layers **(** **Figure 2** **, bottom panel;** **Figure 3** **)**. The average neuron soma size in the magnocellular layers (layers 1 and 2) of the LHON LGN was smaller than the control LGN (41.9% decrease). Also, the average neuron soma size in parvocellular layers of the LHON tissue was smaller than the average found in any layer of the control LGN (44.7% decrease). However, because the relative magnitude of the cell size decrease was similar across all six layers of the LHON LGN, the ratio between the cell size of the magnocellular and parvocellular layers was similar in both the LHON (1.51) and control (1.44) LGNs **(** **Table 2** **)**. No consistent differences were noted in cell size between the nasal, middle (corresponding to the papillomacular bundle), or temporal zones or individual layers. Additionally, the average neuron density of the LHON LGN was decreased across all layers **(** **Table 2** **)**. The two magnocellular layers of the LHON LGN had a reduced average density by 28.5%, and the parvocellular layers of the LHON LGN had a reduced average density by 28.7% when compared with the control LGN.

**Table 2:** Top: neuron soma size by layer type for LHON and control LGN.

Lateral geniculate nucleus: cell body size by layer type					
Layers	Control		LHON		% decrease
1–2 (magnocellular)	388.54 µm^2^		225.68 µm^2^		41.92%
3–6 (parvocellular)	269.77 µm^2^		149.22 µm^2^		44.69%
Ratio	1.44		1.51		/
**Lateral geniculate nucleus: cell density by layer type**					
**Layers**	**Control**		**LHON**		**% decrease**
1–2 (magnocellular)	185.74 cells/mm^2^		132.76 cells/mm^2^		28.52%
3–6 (parvocellular)	263.46 cells/mm^2^		187.80 cells/mm^2^		28.72%
**Optic nerve: axonal counts**					
**Control**		**LHON**		**% decrease**	
993,762		8,200		99.17%	

The ratio of the magnocellular to parvocellular layers for the two LGN is similar suggesting that the atrophy seen in the LHON case was consistent across all layers. Middle: average cell density of the magnocellular and parvocellular layers for both the LHON and control LGNs. LHON LGN exhibits a decrease in neuron density consistent across both cell layer types. Bottom: axonal counts for LHON and control left optic nerve.

The post-mortem retrobulbar optic nerve of the LHON patient stained with PPD showed a dramatic reduction in axonal profiles, which were counted as 8,200 in the left eye (over 99% decrease) as compared to the 993,762 axons counted in the age-matched control (**Figure 2** **, top panel**). The counts of this age-matched control were consistent with previously reported axonal counts of normal individuals in their 70s [23].

## Discussion

In this study we have demonstrated increased diffusivity in the optic radiations (OR) of patients with LHON using diffusion-weighted imaging (DWI). No differences were detected for diffusivity values in the prefrontal and cerebellar white matter. These results confirm the previous observation of post-geniculate abnormalities in LHON patients [12], [13]. DWI changes in OR of LHON affected patients were more severe in those who failed to recover visual acuity and had longer disease duration, whereas they were not detected in unaffected LHON mutation carriers. Furthermore, pathological analysis of the LGN showed three important findings: i) LHON LGN atrophy as evidenced by the significant decrease in the average neuron cell size; ii) LHON LGN degeneration shown by a decrease in neuron density; iii) the changes were consistent across all layers of the LGN as the percent decrease in density was the same for both magnocellular and parvocellular layers, with a similar ratio between the magnocellular and parvocellular layers either in the LHON as in control LGNs.

A previous diffusion-weighted study in LHON found no post-geniculate changes, but this was probably related to the small sample size (ten patients) and the smaller ROI size used [24]. Conversely, another magnetisation transfer (MT) imaging and DWI study from the same group [25] reported significantly lower MT ratio histogram peak height and a trend towards significant increase of average diffusivity values in LHON patients, using histogram analysis of the whole normal appearing white matter, a technical approach with greater statistical power [11]. The authors interpreted these findings as a reflection of the tissue loss and disorganization in the visual pathway or of diffuse and microscopic brain pathology. Both hypotheses might explain the small change in the histograms. Our data corroborate the first hypothesis disclosing changes only in the ORs and not in the extra-visual white matter. Furthermore, two more recent imaging studies documented the specific post-geniculate involvement in LHON patients [12]–[13]. A VBM study demonstrated significant reduced grey matter volume in the bilateral primary visual cortex, and reduced white matter volume in several areas located in the optic radiations, bilaterally [12]. The same group, using diffusion tensor imaging, reported significant diffusivity abnormalities at level of the OR of LHON patients [13]. These studies, unlike the present study, failed to disclose a correlation with disease duration, probably due in part to their inferior statistical power (considering 12 [12] and 13 [13] patients respectively, compared to the 22 of the present study) and the different statistical analysis employed (single correlations [12]–[13] vs the GLM model in our study). These studies did not clarify whether post-geniculate damage in LHON is due to trans-synaptic degeneration or mitochondrial dysfunction. Indeed, previous ^31^P MRS studies detected metabolic abnormalities in the occipital white matter lobes from both LHON patients and healthy carriers [9], [14]–[16]. These observations may suggest that the structural changes in OR were primary and related to degenerative changes due to the mitochondrial dysfunction. On the other hand, our results showed an absence of DWI alterations in LHON carriers and an increase of MD values more evident in LHON patients with longer disease duration and lack of recovery of visual acuity, who are known to have fewer optic nerve fibers compared with LHON patients who recovered [22]. Moreover, extra-visual LHON white matter was not affected. These observations suggest that in LHON patients, involvement of the posterior visual pathways is secondary to trans-synaptic atrophy or degeneration.

Trans-neuronal or trans-synaptic degeneration consists of atrophy or degeneration of post-synaptic neurons deprived of their afferents and has been described in a variety of neural systems [26]. In the visual system, such evidence of dysfunction of the posterior visual pathways, i.e. LGN, OR and calcarine cortex, has been reported as secondary to loss of retina and/or optic nerve after enucleation of one eye [27], chronic glaucoma [28], [29], retinal degeneration [30], and optic neuritis [31]. Concerning optic neuritis and multiple sclerosis, the relevance of trans-synaptic degeneration is currently debated, with particular reference to the grey matter involvement [31], [32], [33]. At the LGN level trans-synaptic changes have been described as neuronal atrophy (cell shrinkage) and, in long-lasting conditions as advanced glaucoma, as neuronal loss [28], [29]. This matches well with our pathological findings in the LGN of a LHON patient with very long disease duration (53 years) and a particularly severe loss of vision (light perception), consistent with axonal loss of over 99% in the optic nerve. We documented mostly neuronal atrophy and, to a lesser extent, some neuronal loss. These changes were consistent across both magno-cellular and parvo-cellular layers of the LGN well fitting the data from trans-neuronal degeneration studies in glaucoma, reporting a different cell loss between magno-cellular and parvo-cellular layers when the RGC axon loss is mild and an equivalent cell loss in all layers when the RGC axon loss is near to 100% (28), as in our LHON patient. Similar results were described for the LGN in old, pre-molecular, post-mortem histopathological studies in a few patients affected by optic atrophy compatible with the LHON diagnosis [34]–[36]. Accordingly, the *in vivo* abnormalities of OR observed by DWI in LHON patients with a shorter disease duration probably reflected neuronal cell and axonal atrophy (shrinkage) secondary to a reduction of synaptic inputs. This suggests that if action potentials could be re-established in the spared axons of the optic nerve, as possibly occurs in those patients recovering visual acuity after the acute phase [1], [2], [4], [5], the recovery of the synaptic inputs might lead to recovery of atrophic but still viable neurons in the LGN and their axons in the optic radiations. Thus, it is possible that the neuron soma shrinkage represents a fairly long therapeutic window of opportunity prior to cell death. The further development of post-geniculate changes with increasing RGC and axonal loss suggests that any effective treatment should be early. Nevertheless, treatment could be useful even in the later stages of the disease, as suggested by the slow recovery of visual acuity, spontaneous or after idebenone treatment in LHON [1], [2], [4], [5]. Unfortunately, we could not study a further group of LHON patients without idebenone therapy in order to investigate its possible influence on DWI parameters. However, it should be noted that idebenone has been shown to increase recovery rate in LHON patients (5), and in our opinion, recovery of visual acuity was the most powerful variable potentially impacting on post-geniculate integrity. Future work should directly investigate this important issue.

A limitation of this study is the use of DWI rather than DTI (diffusion tensor imaging), not available at our institution when the study was started, that precluded the measurement of other parameters such as fractional anisotropy, parallel diffusion, and radial diffusion, which might have given more information about the type of degeneration occurring in the long white matter tracts [37]. Interestingly, the previous DTI study detected decreased fractional anisotropy and an increased radial diffusivity in the OR of LHON patients [13], consistent with the DTI changes observed in the chronic stages of secondary axonal degeneration [38]. At most the use of DWI should have reduced the sensitivity of the analyses, creating a bias towards negative results. The limitation of our use of DWI is partially mitigated by the integration with the pathological analysis of LGN that provided an independent confirmation on the changes detected. The latter, despite being performed in a single case showed histopathological changes that were very similar to other descriptions of LGN histopathology performed in LHON patients prior to the availability of molecular diagnosis [34]–[36].

A further relative limitation of our study is the lack of retinal nerve fiber layer (RNFL) data from optical coherence tomography investigations (OCT) for use in the correlation analysis, as not all our patients had undergone OCT while others with available OCT had undergone the examination too far from the MR acquisition. The finding of a correlation between RNFL data and OR changes would further support the transneuronal hypothesis. Notably, such a correlation was found in the previously cited VBM study [12]. In conclusion, this study extends the results of recent similar investigations, by using a larger group of well-characterized LHON patients and adding the rare opportunity to verify on a single post-mortem specimen the MR finding at the histological level. Using DWI, we detected microstructural changes in ORs and not in extra-visual white matter of LHON affected patients. This was more evident in LHON patients with longer disease duration and absence of recovery of visual acuity. In contrast, the unaffected LHON carriers showed no changes despite previous evidence of metabolic impairment in the same pathway. Pathological examination of the LGN from the single separate LHON patient with very severe optic atrophy suggested that these microstructural changes were mainly due to neuronal and axonal atrophy of the post-synaptic cell. Thus, the post-geniculate involvement in LHON patients is most likely a downstream secondary phenomenon, from chronic de-afferentation, rather than mitochondrial dysfunction associated with primary neurodegeneration.
